# Characterizing the role of Phlda3 in the development of acute toxicity and malignant transformation of hematopoietic cells induced by total-body irradiation in mice

**DOI:** 10.1038/s41598-023-39678-2

**Published:** 2023-08-09

**Authors:** Stephanie Hasapis, Isibel Caraballo, Timothy J. Sears, Kennedy D. Brock, John B. Cart, Everett J. Moding, Chang-Lung Lee

**Affiliations:** 1 Department of Radiation Oncology, Duke University School of Medicine, Duke University Medical Center, Box 3813, Durham, NC 27708 USA; 2 Department of Pathology, Duke University School of Medicine, Durham, NC 27710 USA; 3 Department of Radiation Oncology, Stanford Cancer Institute, Stanford University, 875 Blake Wilbur Drive, Stanford, CA 94305-5847 USA; 4 Stanford Cancer Institute, Stanford University, Stanford, CA USA

**Keywords:** Cancer, Cancer genomics, Haematological cancer, Tumour-suppressor proteins, Oncogenesis, Haematological cancer, Cell biology, Cell death

## Abstract

The tumor suppressor p53 is a transcriptional factor that plays a crucial role in controlling acute toxicity and long-term malignant transformation of hematopoietic cells induced by genotoxic stress such as ionizing radiation. Among all transcriptional targets of p53, one gene that is robustly induced by radiation is the pleckstrin homology domain-only protein Phlda3. However, the role that Phlda3 plays in regulating the response of hematopoietic cells to radiation is unknown. Here, using isogenic cell lines and genetically engineered mouse models, we showed that radiation induces *Phlda3* in human leukemia cells and mouse normal hematopoietic cells in a p53-dependent manner. However, deletion of the *Phlda3* gene did not ameliorate radiation-induced acute hematologic toxicity. In addition, distinct from mice that lose *p53*, loss of *Phlda3* did not alter the latency and incidence of radiation-induced thymic lymphoma in mice. Remarkably, whole-exome sequencing data showed that lymphomas in irradiated *Phlda3*^+*/*+^ mice harbor a significantly higher number of single nucleotide variants (SNVs) and indels compared to lymphomas in irradiated *Phlda3*^+*/−*^ and *Phlda3*^*−/−*^ littermates. Together, our results indicate that although deletion of *Phlda3* does not accelerate the development of radiation-induced thymic lymphoma, fewer SNVs and indels are necessary to initiate lymphomagenesis after radiation exposure when *Phlda3* is silenced.

## Introduction

Hematopoietic cells are one of the most sensitive cell types to ionizing radiation^1^. Total-body irradiation (TBI) can cause acute toxicity and long-term malignant transformation of hematopoietic cells in mice and humans. Numerous studies demonstrate that one key protein that controls the response of hematopoietic cells to radiation is the tumor suppressor p53^2,3^. Induction of p53 following irradiation in leukocytes and hematopoietic stem/progenitor cells (HSPCs) activates the intrinsic pathways of apoptosis through a variety of transcriptional targets^4,5^. For example, Puma (p53 up-regulated mediator of apoptosis) is a transcriptional target of p53 that has been shown to play a key role in regulating radiation-induced apoptosis of hematopoietic cells^6–8^. Hematopoietic cells in both *Puma*^+*/−*^ and *Puma*^*−/−*^ mice are resistant to apoptosis induced by radiation compared to hematopoietic cells in *Puma*^+*/*+^ mice^9,10^. In addition, deletion of *Puma* significantly protects mice from the development of hematopoietic acute radiation syndrome (H-ARS)^9,10^.

As one of the most important tumor suppressors in mammalian cells, p53 is also essential to prevent the development of hematologic malignancies induced by ionizing radiation. For example, mice that lose one allele of the wild type p53 (p53^+/−^) are highly susceptible to the development of thymic lymphoma following TBI^11,12^. However, mechanisms underlying this phenomenon remain incompletely understood. Previous studies demonstrated that mice lacking *Puma* or *Noxa*, both of which are transcriptional targets of p53 that regulate apoptosis, show distinct susceptibility to radiation-induced thymic lymphoma^13^. While deletion of *Noxa* accelerates thymic lymphoma formation after irradiation, loss of *Puma* protects mice from thymic lymphoma induced by TBI^13^. These findings indicate the complex role by which p53-mediated signaling regulates malignant transformation of hematopoietic cells induced by radiation. However, although numerous transcriptional targets of p53 have been identified in response to genotoxic and/or oncogenic stress^14^, only a small subset of these genes have been studied regarding their roles in regulating the radiation response of hematopoietic cells in vivo.

Here, we aimed to study the role of Phlda3 (pleckstrin homology-like domain, family A, member-3) in regulating the development of acute hematologic toxicity and hematopoietic malignancies induced by total-body irradiation in mice. Phlda3 (also called Tih1) is a member of the PHLDA gene family including Phlda1, Phlda2 and Phlda3. The *Phlda3* gene has been identified as a direct transcriptional target of p53^14–16^. Experiments using the NCI-60 human cancer cell lines demonstrate that *Phlda3* is one of the most consistently induced genes by ionizing radiation among p53 wild type cancer cells^17^. Several papers reveal that Phlda3 is involved in various signaling pathways in mammalian cells including Akt signaling^15,18,19^ and endoplasmic reticulum stress^20,21^. In addition, experiments using mice that harbor mutations in different transactivation domains of p53 suggest Phlda3 is a putative tumor suppressor^14^. In this study, we performed a series of experiments using genetically engineered mice that lack *Phlda3* in the germline (*Phlda3*^*−/−*^) to investigate the role of Phlda3 in regulating acute and long-term effects of ionizing radiation on hematopoietic cells in vivo.

## Materials and methods

### Mouse strains and procedures

All animal procedures for this study were approved by the Institutional Animal Care and Use Committee (IACUC) at Duke University (protocol number A215-20-11 was approved on November 9, 2020). All methods were performed in accordance with the relevant guidelines and regulations. The study is reported in accordance with ARRIVE guidelines. Mice with germline deletion of *Phlda3* (*Phlda3*^*−/−*^ or called *Tih1*^*−/−*^ in the original publication)^22^ were kindly provided by Dr. Benjamin Tycko’s laboratory and have been maintained on a C57BL/6J background (CD45.2). In this mouse strain, exon 1 of the *Phlda3* gene is replaced by a neomycin cassette^22^. B6.SJL mice (CD45.1) were purchased from The Jackson Laboratory (#002,014). *Tie2Cre* mice were crossed to *Rosa26-LSL-mp53*^*R172H*^ mice (The Jackson Laboratory Strain #:026,283) to generate mice that express a mutant p53^R172H^ protein in hematopoietic cells^23,24^. *Tie2Cre; p53*^*FL/−*^ mice were characterized previously^23^. *Phlda3*^+*/*+^, *Phlda3*^+*/−*^ and *Phlda3*^*−/−*^ littermates around 4 weeks old were used for radiation-induced thymic lymphoma experiments, whereas in other experiments mice around 8 to 12 weeks old were used. Both male and female mice were used for experiments. Mouse genotyping was performed by Transnetyx.

For the acute radiation syndrome experiments, the health status of irradiated mice was monitored by trained laboratory personnel at least 3 times per week. No supportive care was given to mice after irradiation. The criteria for euthanasia were based on two parameters: decreased activity and squinted/closed eyes on a scale of 1–3 as previously described^25^. A mouse receiving a score of 3 in either category was considered moribund and was euthanized by carbon dioxide asphyxiation. To perform complete blood count (CBC), submandibular cheek bleed was performed to collect whole blood into EDTA tubes (Sarstedt). The samples were then run for CBC by the Duke Veterinary Diagnostic Laboratory.

### Radiation treatment

Total-body irradiation was performed 50 cm from the radiation source with a dose rate of 204 cGy/min with 320 kVp X-rays, using 12.5 mA and a filter consisting of 2.5 mm Al and 0.1 mm Cu (X-RAD 320 Biological Irradiator, Precision X-ray). The dose rate was measured with an ion chamber by members of the Radiation Safety Division at Duke University.

### Gene expression analysis

RNA was isolated from thymic tissues using Zymo Direct-zol RNA Miniprep Kit or Zymo Quick-RNA MiniPrep Plus Kit (Zymo Research) according to the manufacturer’s protocols. cDNA was synthesized using iScript cDNA Synthesis Kit (Bio-Rad). Gene expression for mouse Phlda3 (Mm00449846_m1) was analyzed using Taqman probes and TaqMan™ Universal PCR Master Mix (ThermoFischer) with Gapdh (Mm99999915_g1) as an internal control. Human gene expression for Phlda1 (Hs00378285_g1), Phlda2 (Hs00169368_m1), and Phlda3 (Hs00385313_m1) were assessed via TaqMan™ Universal PCR Master Mix with Gapdh (Hs02758991_g1) as an internal control.

### Western blotting

Proteins were extracted from cells using Pierce RIPA Buffer (Thermo Scientific) titrated to 1% SDS (Bio-Rad) with the addition of Halt Protease Inhibitor Cocktail (Thermo Scientific) and Benzonase Nuclease (Sigma). The protein concentration was determined using Pierce Rapid Gold BCA Protein Assay (Thermo Scientific) according to the manufacturer’s protocol. Total protein (35ug) was loaded for electrophoresis into 4–20% sodium dodecyl sulfate polyacrylamide gels (Bio-Rad). Separated proteins were transferred to a 0.22 µm nitrocellulose membrane (Bio-Rad). Membranes were blocked with Odyssey Blocking Buffer (Li-Cor) diluted 1:1 with tris buffered saline with 0.1% Tween 20 (TBS-T). Membranes were then cut into different strips and stained with antibodies against GAPDH (D16H11 Cell Signaling Technology #5174, 1:1000 dilution), p53 (7F5, Cell Signaling Technology #2527, 1:1000 dilution), PHLDA3 (Cell Signaling Technology #4294, 1:1000 dilution), and p21 Waf1/Cip1 (12D1 Cell Signaling Technology #2947, 1:1000 dilution) followed by secondary fluorescently-conjugated IRDye 800CW (Li-Cor, 1:20,000 dilution). Bands were visualized at 700 and 800 nm using the Odyssey CLX imaging system (Li-Cor).

### Detection of thymocyte cell death in vitro

Thymocytes were isolated following the procedure described previously^3^. Isolated thymocytes were cultured in RPMI 1640 medium supplemented with 25 mM HEPES, 10% fetal bovine serum and penicillin/streptomycin. Irradiation was conducted about 1 h after cell culture in vitro. At 24 h after irradiation, thymocytes were collected, washed using PBS supplemented with Ca^2+^ and Mg^2+^, and then stained with a FITC-conjugated annexin V (BD Pharmigen) and propidium iodide (5 μg/ml, Sigma-Aldrich). Data were collected from at least 20,000 cells by FACSCanto (BD Pharmingen) and analyzed by Flowjo (Tree Star, Inc) without knowledge of the genotype or treatment by a single observer (C-LL).

### Quantification of colony-forming cells

Whole bone marrow cells were harvested following the procedure described previously^3^. 2 × 10^4^ whole bone marrow cells were plated in MethoCult GF 3434 (Stem cell technologies). Whole bone marrow cells were irradiated about 1 h after plating and the number of colonies was counted 7 days later. A colony is defined as clusters with a minimal of 30 cells. Colonies were counted without knowledge of the genotype or treatment by two observers (SH and C-LL).

### Flow cytometry analysis of hematopoietic stem/progenitor cells in the bone marrow

HSPCs were harvested from the bone marrow of unirradiated and irradiated mice and then stained with antibody cocktails described previously^3,26^. Cells were blocked with a rat anti-mouse CD16/32 antibody (BD Pharmingen) and stained with PE-Cy5 conjugated lineage cocktail containing anti-mouse CD3, CD4, CD8, B220, CD11b, Gr-1 and Ter-119 antibodies (eBioscience), anti-mouse CD27 conjugated to APC (Thermo Fisher) and anti-mouse CD201 conjugated to PE (eBioscience). Data were collected from at least 500,000 single cells by FACSCanto (BD Pharmingen) and analyzed by FlowJo (Tree Star, Inc) without knowledge of the genotype or treatment by a single observer (C-LL).

### Competitive repopulation assay

*Phlda3*^+*/*+^ and *Phlda3*^*−/−*^ littermates (CD45.2) 35 days after 2.5 Gy TBI were used as donors for the competitive repopulation assay. 2 × 10^5^ or 1 × 10^6^ whole bone marrow cells from these mice were mixed with 2 × 10^5^ whole bone marrow cells from unirradiated B6.SJL (CD45.1) mice. The mixed bone marrow cells were transplanted into 8-week-old B6.SJL (CD45.1) recipients 6 h after two fractions of 4.75 Gy TBI with an interval of about 18 h. At 16 weeks after bone marrow transplantation, peripheral blood mononuclear cells (PB-MNCs) were harvested from mice following the procedure described previously^3^. PB-MNCs were blocked with a rat anti-mouse CD16/32 antibody (BD Pharmingen) and stained with an antibody cocktail including PE-Cy5 conjugated anti-mouse CD3e (clone: 145-2C11), PE conjugated anti-mouse B220 (clone: RA3-6B2), APC conjugated anti-mouse CD11b (clone: M1/70), FITC conjugated anti-mouse CD45.2 (clone: 104), and APC-eFluor780 conjugated anti-mouse CD45.1 (clone: A20) antibodies (eBioscience). All antibodies were diluted 1:400. Data were collected from at least 20,000 single cells by FACSCanto (BD Pharmingen) and analyzed by FlowJo (Tree Star, Inc) without knowledge of the genotype or treatment by a single observer (C-LL).

### Cell culture

Isogenic MOLM13 cells that express wild-type p53, a p53^R175H^ mutant protein (p53^R175H/−^) or no p53 (p53^−/−^) were generated and kindly provided by Dr. Benjamin L. Ebert’s laboratory^27^. MOLM13 cells were cultured in RPMI Medium 1640 (Gibco 11875-093) supplemented with 10% FBS (Gibco 10082147), 1% Sodium Pyruvate (Gibco 11360070), and 1% Antibiotic–Antimycotic (Gibco 15240062). Cells were maintained at a culturing density between 2.5 × 10^5^ and 2 × 10^6^ cells/mL. For radiation experiments, MOLM13 cells were seeded at 2 × 10^5^ cells/mL before immediate irradiation. Cells were then collected by snap freezing in LN2 at the desired timepoint before storing at − 80 °C. Harvested mouse bone marrow cells and thymocytes were plated at a density of 2 × 10^6^ cells/mL in IMDM (Gibco #12,440,053) supplemented with 1% Antibiotic–Antimycotic and 20% FBS. These cells were then irradiated immediately after plating. Cells were collected at the desired timepoint by snap freezing in LN2 before storing at − 80 °C.

### Detection of thymocyte apoptosis in vitro

Thymocytes were isolated following the procedure described previously^3^. Irradiation was conducted about 1 h after cell culture in vitro. At 24 h after irradiation, thymocytes were collected, washed using PBS supplemented with Ca^2+^ and Mg^2+^, and then stained with a FITC-conjugated annexin V (BD Pharmigen) and propidium iodide (5 μg/ml, Sigma-Aldrich). Data were collected from at least 20,000 cells by FACSCanto (BD Pharmingen) and analyzed by Flowjo (Tree Star, Inc) without knowledge of the genotype or treatment by a single observer (C-LL).

### Sample preparation for whole exome sequencing

To prepare samples for WES, thymic lymphoma specimens stored in RNAlater (Invitrogen) and matched snap frozen tails were used for DNA extraction. DNA extraction was performed using the DNeasy Blood and Tissue Kit or the AllPrep DNA/RNA Mini Kit (Qiagen) per the manufacturer’s guidelines. Hybrid capture was performed according to the manufacturer’s instructions using the Twist Mouse Exome Panel (Twist Bioscience) to enrich for 37.7 megabases targeting the mouse exome. Enriched libraries were sequenced on an Illumina NovaSeq 6000 S1 lane with 150 bp paired-end reads. Samples were demultiplexed and FASTQ files were generated using Bcl2Fastq2 (Illumina).

### Whole exome sequencing data analysis

Sequencing data were processed according to the Genome Analysis Toolkit best practices to identify single nucleotide variants (SNVs), small insertions and deletions (indels), and somatic copy number alterations (SCNAs)^28^. Raw sequencing data was aligned to the mm10 mouse genome assembly using BWA-ALN (v0.5.9-r16)^29^. Aligned BAM files were preprocessed using Picard tools (v4.1), and somatic SNVs and indels were called from the paired lymphoma and normal samples using Mutect2^30^. SNVs were filtered to remove variants (1) located in repetitive regions of the genome (http://www.repeatmasker.org), (2) with more than 1 alternative read in the matched normal sample, (3) with fewer than 2 alternative reads in the lymphoma sample, and (4) an allele fraction of less than 5%. The Ensembl Variant Effect Predictor (VEP) was used to estimate the impact of SNVs and indels on protein function^31^. SCNAs were identified using CNVkit to integrate on-target and off-target sequencing reads^32^. All normal samples were used as a pooled background reference. SCNAs were filtered to remove copy number variants: (1) present within the matched normal sample and (2) with an absolute log_2_ copy number ratio less than 0.2. For plotting, cancer-driving genes were defined based on the Catalogue of Somatic Mutations in Cancer (COSMIC) Cancer Gene Census (CGC)^33^.

### Statistics

Data are presented as mean ± SEM. Student’s *t*-test (two-tailed) was performed to compare the means of two groups, and one-way ANOVA was performed to compare the means of three or more groups. For survival studies, Kaplan–Meier analysis was performed followed by the log-rank test. To analyze WES data, Two-sided Fisher’s exact tests were used to compare the proportion of lymphomas from each genotype with SNVs/indels or SCNAs in each Tier 1 COSMIC gene. Only genes altered in at least 1 lymphoma were included in the analysis, and P-values were corrected for multiple hypothesis testing using the Benjamini–Hochberg procedure. The total number of each type of mutation was compared across lymphomas from mice of each genotype using Kruskal–Wallis tests followed by Dunn’s tests for pairwise comparisons. Statistical significance was assumed at *P* < 0.05. Statistical analyses were performed with Prism 9 (GraphPad Software) or R version 4.1.2 through the RStudio environment.

## Results

### Induction of Phlda3 by ionizing radiation in hematopoietic cells is p53-dependent

**Figure 1:**
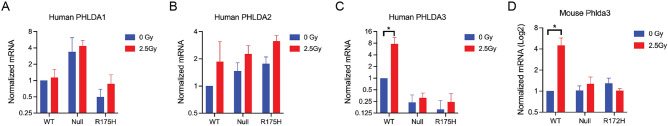
Ionizing radiation induces *Phlda3* mRNA in hematopoietic cells in a p53-dependent manner. (**A**–**C**) Isogenic Human MOLM13 acute myeloid leukemia cells that express wild type p53 (WT), no p53 (null) or a p53^R175H^ mutant protein (R175H) were exposed to 0 or 2.5 Gy X-rays. Cells were harvested 2 h after irradiation to detect the expression of *Phlda1*, *Phlda2* and *Phlda3* mRNA. Data are presented as mean ± SEM. N = 3 independent experiments per group. *P < 0.05 by Student’s t-test compared to 0 Gy. (**D**) Whole bone marrow cells harvested from mice expressing wild type p53 (WT), no p53 (null) or a p53^R172H^ mutant protein (R172H) were exposed to 0 or 2.5 Gy X-rays. Cells were harvested 4 h after irradiation to detect the expression of *Phlda3* mRNA. Data are presented as mean ± SEM. N = 3 to 4 mice per genotype. *P < 0.05 by Student’s t-test compared to 0 Gy.

To determine the role of p53 in controlling the induction of PHLDA family genes in human and mouse hematopoietic cells following ionizing radiation, we first examined mRNA expression of *PHLDA1*, *PHLDA2* and *PHLDA3* in isogenic human MOLM13 acute myeloid leukemia cells that harbor wild type p53 (p53^+/+^), no p53 (p53^−/−^) or a p53^R175H^ mutant protein (p53^R175H/−^) after exposure to 0 or 2.5 Gy X-rays. This radiation dose was selected because it has been shown that radiation doses of 2.5 Gy or less induce strictly p53-dependent cell death^34,35^. Our results showed that 2.5 Gy significantly induced the expression of *PHLDA3* mRNA in MOLM13 p53^+/+^ cells 2 and 4 h post-irradiation, whereas the induction was abrogated in p53^−/−^ and p53^R175H/−^ counterparts. In contrast, the expression of *PHLDA1* and *PHLDA2* was not significantly induced by 2.5 Gy in any of the p53^+/+^, p53^−/−^ or p53^R175H/−^ MOLM13 cells (Fig. 1A–C and Figure S1). Of note, the expression of PHLDA3 protein was below the detection level, while the protein expression of p53 and p21, a canonical transcriptional target of p53^36^, was induced by 2.5 Gy only in MOLM13 p53^+/+^ cells (Figure S2). We also examined mRNA expression of *Phlda3* in whole bone marrow cells of *Tie2Cre; p53*^*FL/−*^ mice, in which both alleles of *p53* are deleted in hematopoietic cells^23^ as well as in *Tie2Cre; Rosa26-LSL-mp53*^*R172H/*+^ mice, which express a p53^R172H^ mutant protein (an equivalent of human p53^R175H^) in the hematopoietic system, and their littermate controls (no Cre; *p53*^+*/*+^). Our data showed that 2.5 Gy significantly induced the expression of *Phlda3* mRNA in bone marrow cells harvested from *p53*^+*/*+^, but not from *Tie2Cre; p53*^*FL/−*^ and *Tie2Cre; LSL-mp53*^*R172H/*+^ mice (Fig. 1D). Together, our findings indicate that among all three PHLDA family genes, *PHLDA3* is the only one that is induced by ionizing radiation via a p53-dependent mechanism in hematopoietic cells of mice and humans at the mRNA level.

### Loss of Phlda3 does not protect hematopoietic cells from radiation

**Figure 2:**
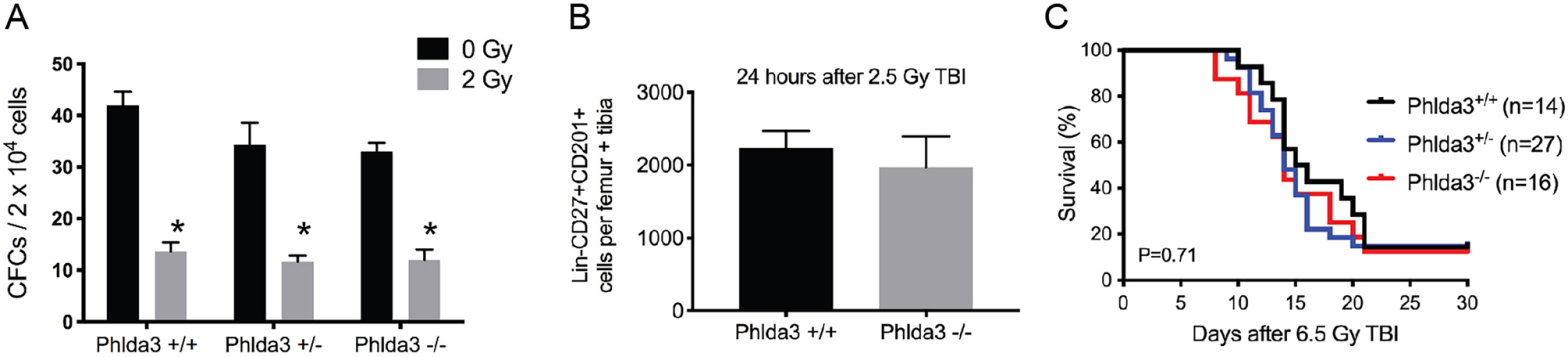
Deletion of *Phlda3* does not ameliorate radiation-induced acute hematopoietic injury. (A) Whole bone marrow cells harvested from *Phlda3*^+*/*+^, *Phlda3*^+*/*−^ and *Phlda3*^*−/−*^ mice were irradiated with 0 and 2 Gy X-rays in vitro. The number of colony-forming cells (CFCs) per 2 × 10^4^ whole bone marrow cells were quantified 7 days after irradiation. N = 3 mice per genotype. Data are presented as mean ± SEM. *P < 0.05 by Student’s t-test compared to 0 Gy. (**B**) Quantification of Lin-CD27 + CD201 + cells in the bone marrow harvested from *Phlda3*^+*/*+^ and *Phlda3*^*−/−*^ mice 24 h after 2.5 Gy TBI. N = 3 mice per genotype. Data are presented as mean ± SEM. (**C**) Overall survival of *Phlda3*^+*/*+^, *Phlda3*^+*/−*^ and *Phlda3*^*−/−*^ mice after 6.5 Gy TBI that causes the hematopoietic acute radiation syndrome. P value was calculated by log-rank test.

To investigate the role of Phlda3 in vivo, we utilized mice in which the *Phlda3* gene is deleted in the germline^22^. All *Phlda3*^+*/*+^*, Phlda3*^+*/−*^ and *Phlda3*^*−/−*^ littermates developed normally and showed no notable difference in complete blood counts (Supplementary Table 1). To determine the impact of Phlda3 loss on the response of HSPCs to radiation, we conducted colony-forming cell (CFC) assays using whole bone marrow cells harvested from *Phlda3*^+*/*+^*, Phlda3*^+*/−*^ and *Phlda3*^*−/−*^ mice. Whole bone marrow cells plated in methylcellulose-based media were irradiated with 0 and 2 Gy X-rays in vitro. Although 2 Gy irradiation significantly reduced the number of CFCs derived from bone marrow cells, loss of Phlda3 did not alter the number of CFCs in vitro (Fig. 2A). In addition, we examined the role of Phlda3 in regulating the radiation response of HSPCs in vivo by assessing the number of Lin^−^CD27^+^CD201^+^ cells^37^. We did not observe a significant difference in the number of Lin^−^CD27^+^CD201^+^ cells in the bone marrow 24 h after 2.5 Gy TBI between *Phlda3*^+*/*+^ and *Phlda3*^*−/−*^ mice (Fig. 2B). In addition, we performed competitive repopulation assays using total bone marrow cells harvested from *Phlda3*^+*/*+^ and *Phlda3*^*−/−*^ littermates 35 days after 2.5 Gy TBI to assess long-term engraftment of irradiated HSPCs^38,39^ (Figure S3A). Our findings from competitive repopulation assays using either a 1:1 or a 5:1 ratio of irradiated (CD45.2) to unirradiated (CD45.1) whole bone marrow revealed that the deletion of *Phlda3* did not significantly improve long-term hematopoietic reconstitution of irradiated HSPCs (Figure S3B and C). Moreover, we examined the role of Phlda3 in the development of H-ARS by exposing *Phlda3*^+*/*+^*, Phlda3*^+*/−*^ and *Phlda3*^*−/−*^ mice to lethal doses of TBI. Our results from the 30-day follow-up did not show significant difference in the overall survival between *Phlda3*^+*/*+^*, Phlda3*^+*/−*^ and *Phlda3*^*−/−*^ littermates after 6.5 Gy TBI (Fig. 2C). In addition to bone marrow cells, we also examined radiation-induced apoptosis of thymocytes harvested from *Phlda3*^+*/*+^, *Phlda3*^+*/−*^ and *Phlda3*^*−/−*^ mice in vitro. Exposure of thymocytes to 2 and 4 Gy X-rays caused a significant increase in the percentage of Annexin V^+^ cells 24 h after irradiation. However, deletion of *Phlda3* did not prevent radiation-induced apoptosis of thymocytes in vitro (Figure S4). Together, these findings demonstrate that although *Phlda3* is induced by radiation in hematopoietic cells, deletion of the *Phlda3* gene does not protect mice from radiation-induced acute hematopoietic injury.

### Loss of Phlda3 does not alter the formation of radiation-induced thymic lymphoma

**Figure 3:**
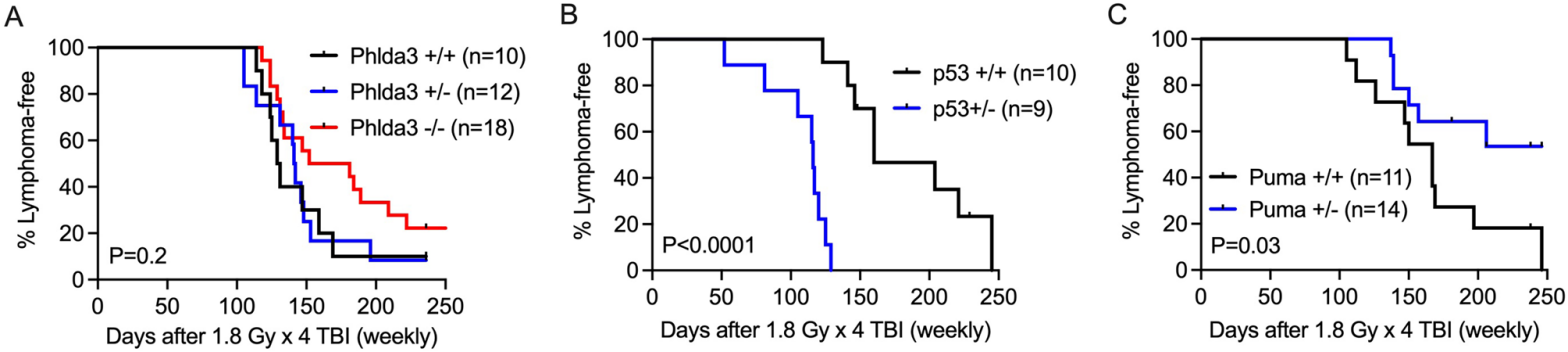
Radiation-induced lymphomagenesis in mice lacking *Phlda3*, *p53* or *Puma*. Radiation-induced thymic lymphoma experiments were conducted using (**A**) *Phlda3*^+*/*+^, *Phlda3*^+*/−*^ and *Phlda3*^*−/−*^ mice, (**B**) *p53*^+*/*+^ and *p53*^+*/−*^ mice and (**C**) *Puma*^+*/*+^ and *Puma*^+*/−*^ mice. Four week old littermates were exposed to 1.8 Gy TBI every week for 4 consecutive weeks (1.8 Gy × 4). The development of thymic lymphoma was followed for up to 250 days after irradiation. *P* values were calculated by log-rank test.

To determine the role of Phlda3 in regulating the development of hematologic malignancies following TBI, we exposed *Phlda3*^+*/*+^*, Phlda3*^+*/−*^ and *Phlda3*^*−/−*^ littermates to 1.8 Gy TBI every week for 4 consecutive weeks (1.8 Gy × 4). This radiation protocol is robust and highly potent to induce thymic lymphoma that resembles human T-cell lymphoblastic lymphoma/leukemia in wild-type mice^40,41^. Exposure to 6 Gy X-rays (a biological equivalent dose close to 1.8 Gy × 4) significantly increased *Phlda3* mRNA expression in *Phlda3*^+*/*+^ and *Phlda3*^+*/−*^ thymocytes 4 h after irradiation (Figure S5). However, our results showed that the incidence and latency of radiation-induced thymic lymphoma were not statistically different among *Phlda3*^+*/*+^*, Phlda3*^+*/−*^ and *Phlda3*^*−/−*^ littermates (Fig. 3A). As the controls for this experiment, we also examined the impact of deleting p53 or Puma on radiation-induced lymphomagenesis. Consistent with published data^11,13,42^, we observed that the development of radiation-induced thymic lymphoma was substantially accelerated in mice with one copy of *p53* (*p53*^+*/−*^) compared to their *p53*^+*/*+^ littermates (Fig. 3B). On the other hand, deletion of *Puma* significantly protected mice from the formation of radiation-induced thymic lymphoma (Fig. 3C). Together, our findings demonstrate that loss of *Phlda3* does not alter the formation of radiation-induced thymic lymphoma in mice.

### Mutational landscape in Phlda3 wild-type and Phlda3 deficient radiation-induced lymphomas

**Figure 4:**
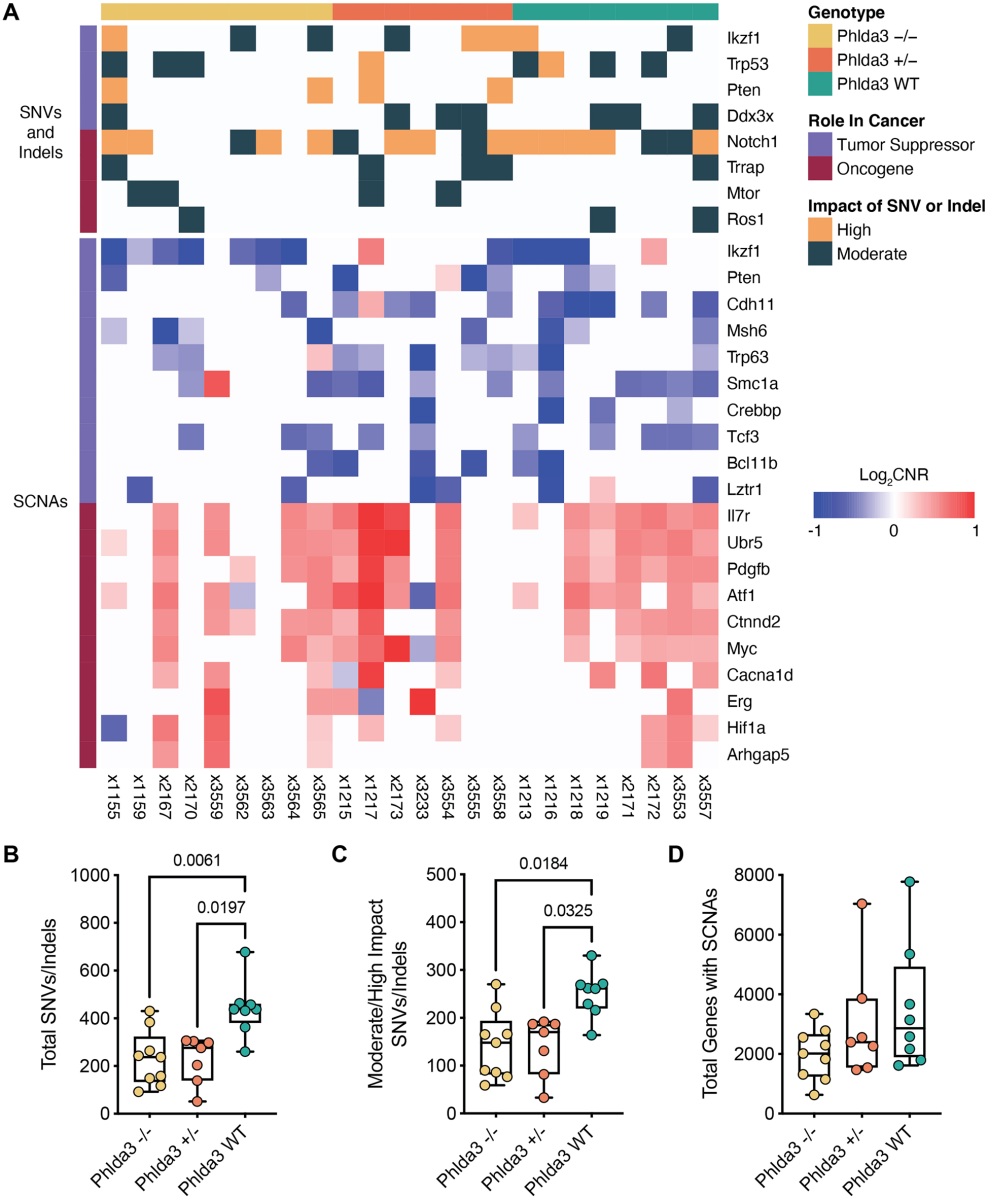
Whole exome sequencing of radiation-induced lymphomas in *Phlda3* wild-type and *Phlda3* deficient mice. (**A**) Plot of genomic alterations identified by whole exome sequencing of radiation-induced thymic lymphomas in *Phlda3*^*−/−*^, *Phlda3*^+*/−*^, and *Phlda3*^+*/*+^ (WT) mice. The top panel shows the tumor suppressors and oncogenes most frequently altered by moderate or high impact SNVs or indels. The bottom panel shows the tumor suppressors and oncogenes with the most frequent SCNAs. Box plots displaying (**B**) the total number of SNVs/indels, (**C**) the number of moderate/high impact SNVs/indels, and (**D**) the total number of SCNAs per lymphoma by mouse genotype. Box shows quartiles and whiskers extend to the minimum and maximum value. *P* values were calculated using Kruskal–Wallis tests followed by Dunn’s tests for pairwise comparisons.

To explore whether loss of *Phlda3* affects the mutational spectrum within radiation-induced thymic lymphomas, we performed whole-exome sequencing of radiation-induced lymphomas from *Phlda3*^*−/−*^, *Phlda3*^+*/−*^, and *Phlda3*^+*/*+^ (WT) mice and identified SNVs, indels, and SCNAs. Consistent with our previous work^40^, we observed frequent activating mutations in the Notch1 pathway across genotypes (Fig. 4A, Supplementary Tables S2 and S3). Of the 24 lymphomas analyzed, 20 (83%) had mutations in *Notch1* or *Ikzf1*. However, there was no significant difference in Notch1 pathway mutations across genotypes. Similarly, we did not observe any significant differences in COSMIC Tier 1 genes affected by SNVs/indels or SCNAs between lymphomas in *Phlda3*^*−/−*^, *Phlda3*^+*/−*^, and *Phlda3*^+*/*+^ mice (Supplementary Tables S4 and S5). Of note, we did not observe Phlda3 mutations in any of the lymphomas.

We next explored whether loss of *Phlda3* affects the number of genomic alterations in radiation-induced lymphomas. We observed significantly more SNVs and indels in *Phlda3*^+*/*+^ compared with *Phlda3*^*−/−*^ and *Phlda3*^+*/−*^ lymphomas (Fig. 4B). When analyzing only SNVs and indels predicted to have a moderate or high impact on protein function, there continued to be significantly more SNVs and indels in *Phlda3*^+*/*+^ mice (Fig. 4C). In contrast, there was no significant difference in the total number of SCNAs across genotypes (Fig. 4D). The fact that both *Phlda3*^*−/−*^ and *Phlda3*^+*/−*^ lymphomas showed a distinct phenotype compared to *Phlda3*^+*/*+^ lymphomas prompted us to examine the expression of *Phlda3* mRNA in these tumors. We observed that *Phlda3* is not expressed in *Phlda3*^*−/−*^ lymphomas, which confirmed the complete loss of the *Phlda3* gene in these tumors. Unexpectedly, we noticed that the expression of *Phlda3* mRNA in *Phlda3*^+*/−*^ lymphomas was approximately 20 folds lower compared to which in *Phlda3*^+*/*+^ lymphomas (Figure S6A). However, SCNA analysis indicated that the substantial decrease in mRNA expression of *Phlda3* in *Phlda3*^+*/−*^ lymphomas cannot be explained by copy number alterations of *Phlda3* (Figure S6B). Together. our results from WES experiments indicate that fewer SNVs and indels are necessary to initiate lymphomagenesis after radiation exposure when *Phlda3* is silenced.

## Discussion

Our data reveal that among three PHLDA family genes, *Phlda3* is the only one that is induced by radiation in human acute myeloid leukemia cells in a p53-dependent manner. This p53-dependent mechanism of Phlda3 induction is also conserved in mouse bone marrow cells (Fig. 1). These findings indicate that Phlda3 is a specific marker of p53 activation in human and mouse hematopoietic cells. Of note, we attempted to detect the expression of PHLDA3 protein in MOLM13 p53^+/+^ AML cells by Western blotting without success (data not shown). These findings suggest the possibility that either the half-life of PHLDA3 protein is short or the level of PHLDA3 protein is below the detection limit. In addition, while our data suggested that *PHLDA1* mRNA was not induced by ionizing radiation in MOLM13 p53^+/+^ AML cells, this PHLDA family gene has been identified as a transcriptional target of p53 in multiple human solid cancer cell lines^43^. Intriguingly, we observed that the expression of *PHLDA1* mRNA at the baseline appears to be even higher in MOLM13 p53^−/−^ AML cells compared to MOLM13 p53^+/+^ AML cells. Thus, it warrants further investigations to elucidate how *PHLDA1* expression is regulated in human AML cells.

Although it has been speculated that induction of Phlda3 would promote p53-dependent apoptosis following radiation in normal tissues, our results demonstrate that deletion of *Phlda3* neither protects bone marrow-derived HSPCs from acute radiation injury nor prevents the development of H-ARS in mice (Fig. 2). One limitation of our study is the use of mice in which *Phlda3* is deleted from the germline because it is possible that hematopoietic cells in *Phlda3*^*−/−*^ mice have adapted the loss of *Phlda3*. This hypothesis could be tested by conducting experiments using mice in which *Phlda3* can be deleted or knocked down in somatic cells acutely before irradiation. Nevertheless, our results indicate that, in contrast to mice with germline deletion of *p53*^2^ or *PUMA*^9,10^, deletion of *Phda3* has very minimal impact, if any, on the response of hematopoietic cells to ionizing radiation in mice.

The tumor suppressor p53 prevents malignant transformation through complex mechanisms including apoptosis, ferroptosis, cell cycle arrest, genomic integrity, metabolism, redox biology, and stemness^44^. One elegant study using mice that harbor mutations in either or both of the transactivation domains of p53 suggested that Phlda3 is a key transcriptional target of p53 that mediates tumor suppression^14^. In addition, it has been reported that Phlda3 is a putative tumor suppressor in pancreatic neuroendocrine tumors^45^. However, using a mouse model of radiation-induced thymic lymphoma, which has been robustly used to study p53-mediated tumor suppression, our results show that deletion of *Phlda3* does not alter the incidence and latency of thymic lymphomas induced by 1.8 Gy × 4 TBI (Fig. 3). While loss of p53 results in a substantial increase in the number of genes affected by SCNA in murine radiation-induced thymic lymphomas^12,40^, SCNA is not significantly different among thymic lymphomas in irradiated *Phlda3*^+*/*+^*, Phlda3*^+*/−*^ and *Phlda3*^*−/−*^ mice. Moreover, we did not detect any somatic mutations in the *Phlda3* gene among all murine thymic lymphomas we examined. Also, examination of human diffuse large B cell lymphoma (DLBCL) dataset from The Cancer Genome Atlas (TCGA) indicate that there are no somatic mutations in *PHLDA3*^46^*.*

Our findings from WES show that lymphomas in irradiated *Phlda3*^+*/*+^ mice harbor a significantly higher number of SNVs and indels compared to lymphomas in irradiated *Phlda3*^+*/−*^ and *Phlda3*^*−/−*^ littermates (Fig. 4). Although mechanisms underlying this phenomenon remain to be further investigated, these data suggest similar processes during thymic lymphomas development between *Phlda3*^+*/−*^ and *Phlda3*^*−/−*^ mice. Indeed, gene expression analysis shows that the expression of *Phlda3* mRNA in *Phlda3*^+*/−*^ lymphomas is not significantly higher than in *Phlda3*^*−/−*^ lymphomas. However, WES data reveal that the suppression of *Phlda3* mRNA in *Phlda3*^+*/−*^ lymphomas cannot be explained by somatic mutations or copy number changes (Figure S6). These results suggest that the wild type allele of Phlda3 in *Phlda3*^+*/−*^ lymphomas is silenced through epigenetic mechanisms. Of note, human pancreatic neuroendocrine tumors that lose one copy of the *Phlda3* allele exhibit frequent promoter hypermethylation of the remaining wild type *Phlda3* gene^45^. Thus, it warrants further investigations to examine alterations of gene-specific and global methylation among thymic lymphomas in irradiated *Phlda3*^+*/*+^*, Phlda3*^+*/−*^ and *Phlda3*^*−/−*^ mice.

Collectively, our findings using *Phlda3* knockout mice demonstrate that loss of *Phlda3* does not alter development of acute hematopoietic injury and thymic lymphoma induced by TBI. However, fewer SNVs and indels are necessary to initiate lymphomagenesis after radiation exposure when *Phlda3* is silenced.

### Supplementary Information

Supplementary Figure 1.

Supplementary Table 2.

## Data Availability

The datasets generated during the current study are publicly available in the NCBI Bioproject repository PRJNA887933 (https://www.ncbi.nlm.nih.gov/bioproject/PRJNA887933).
