# Supplementary material for: Characterizing the role of Phlda3 in the development of acute toxicity and malignant transformation of hematopoietic cells induced by total-body irradiation in mice
Source: Sci Rep. 2023 Aug 9;13:12916. doi: 10.1038/s41598-023-39678-2 (PMC10412554; doi:10.1038/s41598-023-39678-2)
Supplement: Supplementary file 1 — Supplementary Figure 1. [file 41598_2023_39678_MOESM1_ESM.docx]

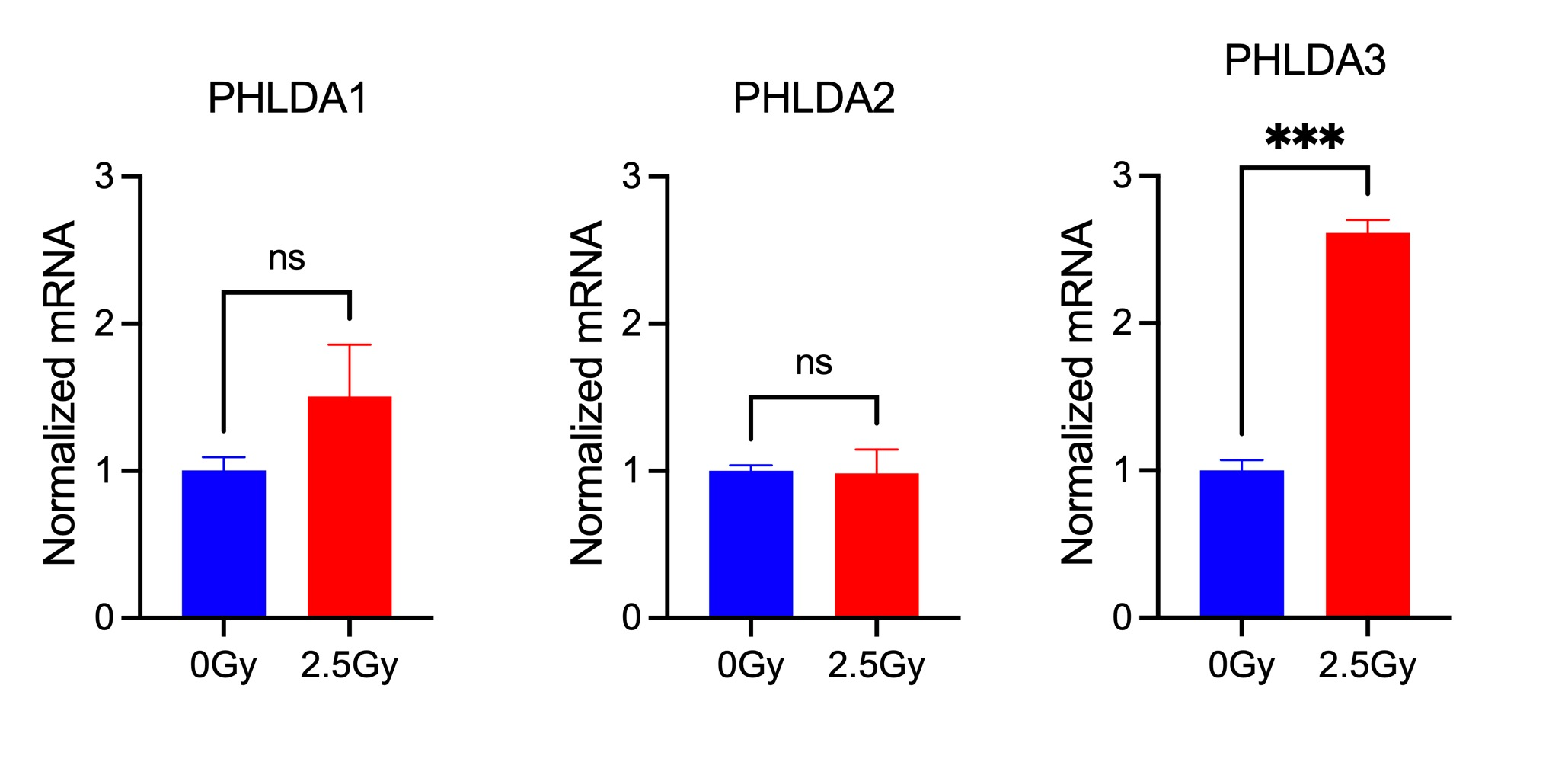


**Figure S1. Ionizing radiation induces *PHLDA3* mRNA in MOLM13 p53^+/+^ cells.** Human MOLM13 cells that express wild type p53 (p53^+/+^) were exposed to 0 or 2.5 Gy X-rays. Cells were harvested 4 hours after irradiation to detect the expression of *PHLDA1*, *PHLDA2* and *PHLDA3* mRNA. Data are presented as mean ± SEM. N=3 independent experiments per group. ***P<0.001 by Student’s t-test compared to 0 Gy.


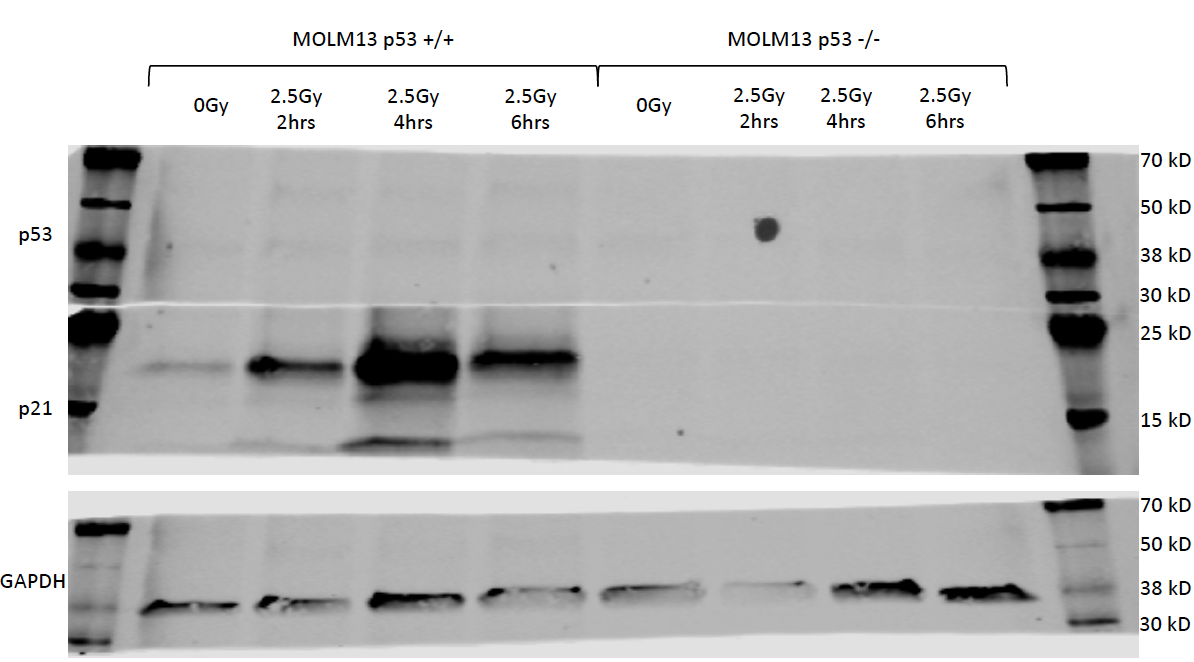


**
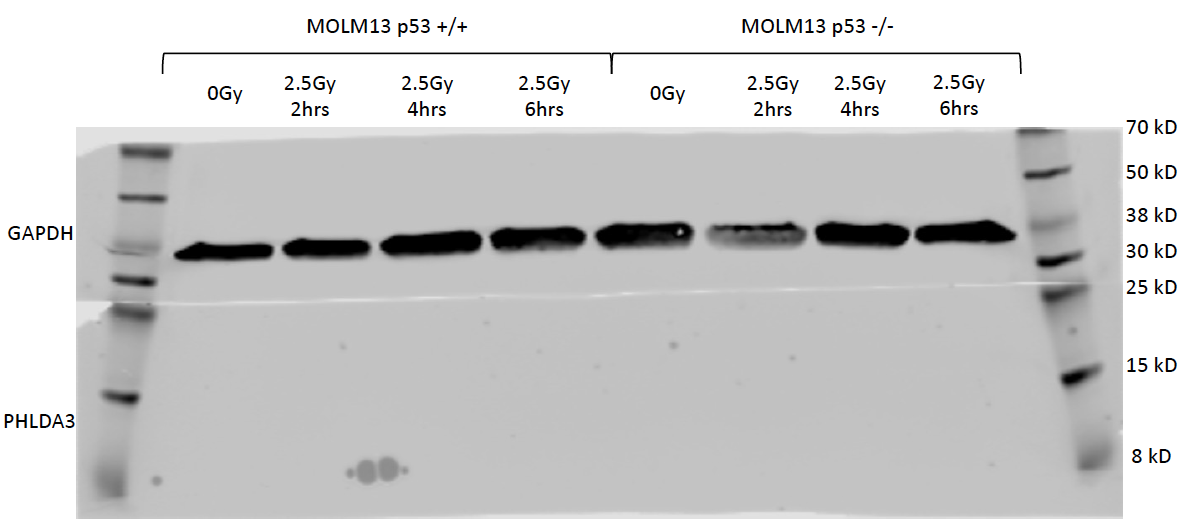
**

**Figure S2.** **Examination the expression of p53, p21, and PHLDA3 proteins in MOLM13 cells with and without irradiation.** Representative Western blotting that examines the expression of p53, p21, and PHLDA3 proteins in MOLM13 p53^+/+^ and MOLM13 p53^-/-^ AML cells without irradiation (0 Gy) or at various time points after 2.5 Gy X-rays. GAPDH was used as a housekeeping gene. The predicted molecular weight of each proteins is: p53 (53 kD), p21 (21 kD), PHLDA3 (15 kD), and GAPDH (37kD). The same amount of protein per sample was loaded for both membranes.


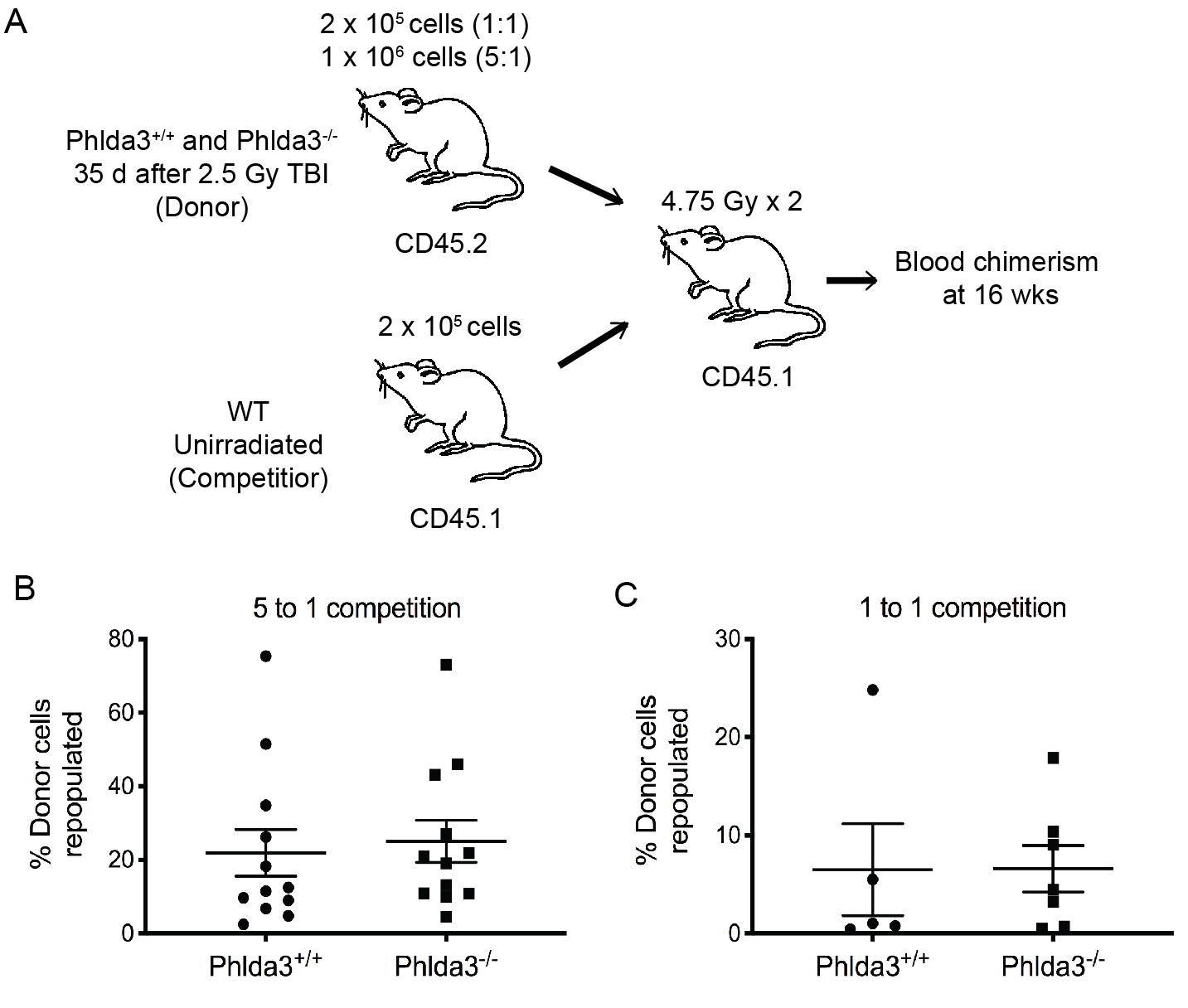


**Figure S3.** **Deletion of Phlda3 does not alter long-term engraftment of irradiated hematopoietic stem/progenitor cells. A)** Schematic representation of the competitive repopulation assay. 2 x 10^5^ or 1 x 10^6^ whole bone marrow cells from Phlda3^+/+^ and Phlda3^-/-^ littermates on a CD45.2 background 35 days after 2.5 Gy TBI were mixed with 2 x 10^5^ whole bone marrow cells from unirradiated B6.SJL (CD45.1) mice. The mixed bone marrow cells were transplanted into lethally irradiated B6.SJL (CD45.1) recipients. The chimerism of CD45.1/2 in the peripheral blood was analyzed 16 weeks after BMT. **B – C)** The percentage of peripheral blood cells repopulated by CD45.2 donors 16 weeks after BMT. Data are presented as mean ± SEM. Each dot represents one recipient mouse.


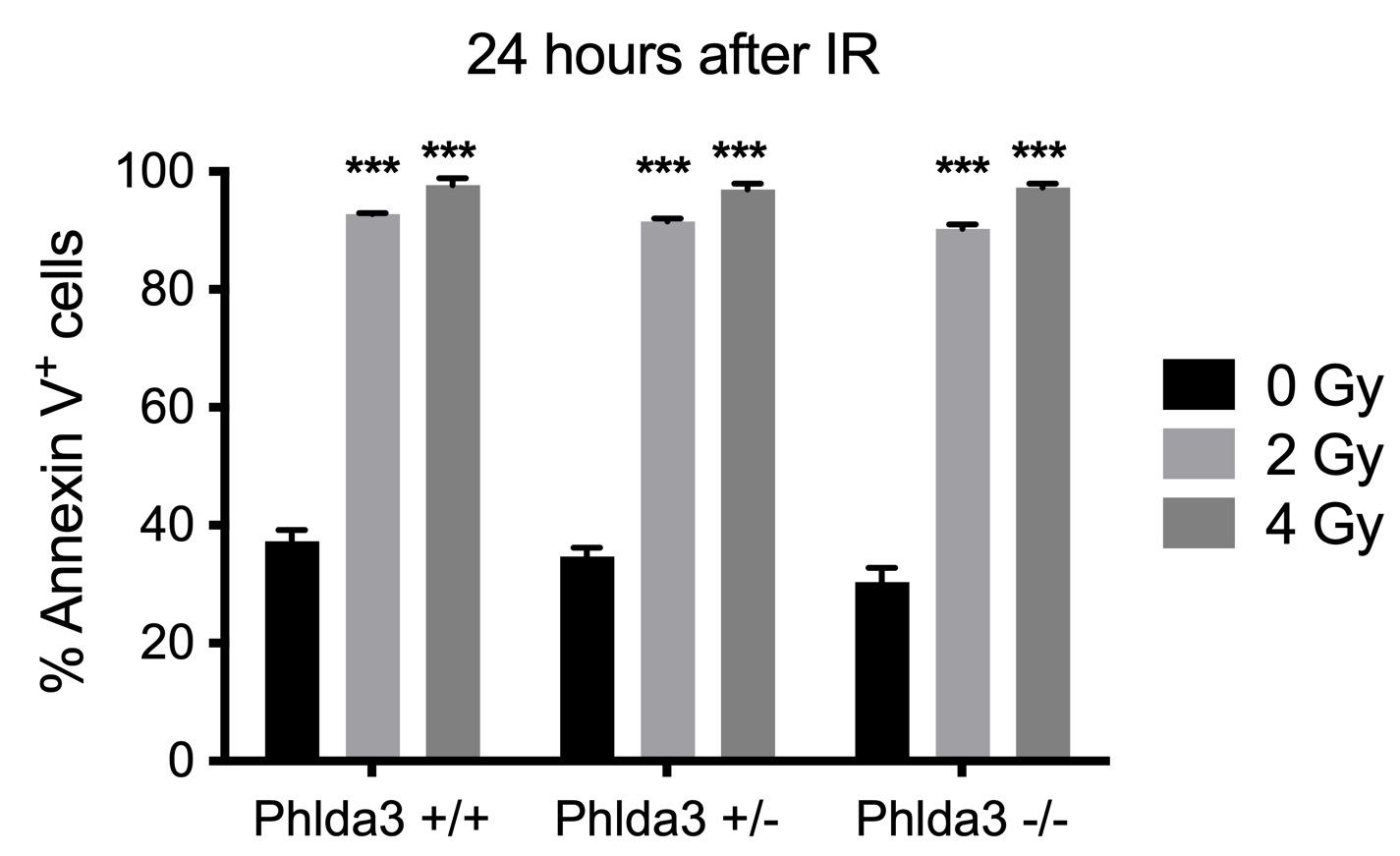


**Figure S4.** **Deletion of *Phlda3* does not alter radiation-induced apoptosis of thymocytes** Thymocytes harvested from *Phlda3^+/+^* , *Phlda3^+/-^* and *Phlda3^-/-^* mice were irradiated with 0, 2 and 4 Gy X-rays *in vitro*. Thymocyte apoptosis was detected by staining with Annexin V-PI 24 hours after irradiation. N=3 independent experiments. Data are presented as mean ± SEM ***p<0.001 by Student’s t-test compared to 0 Gy.


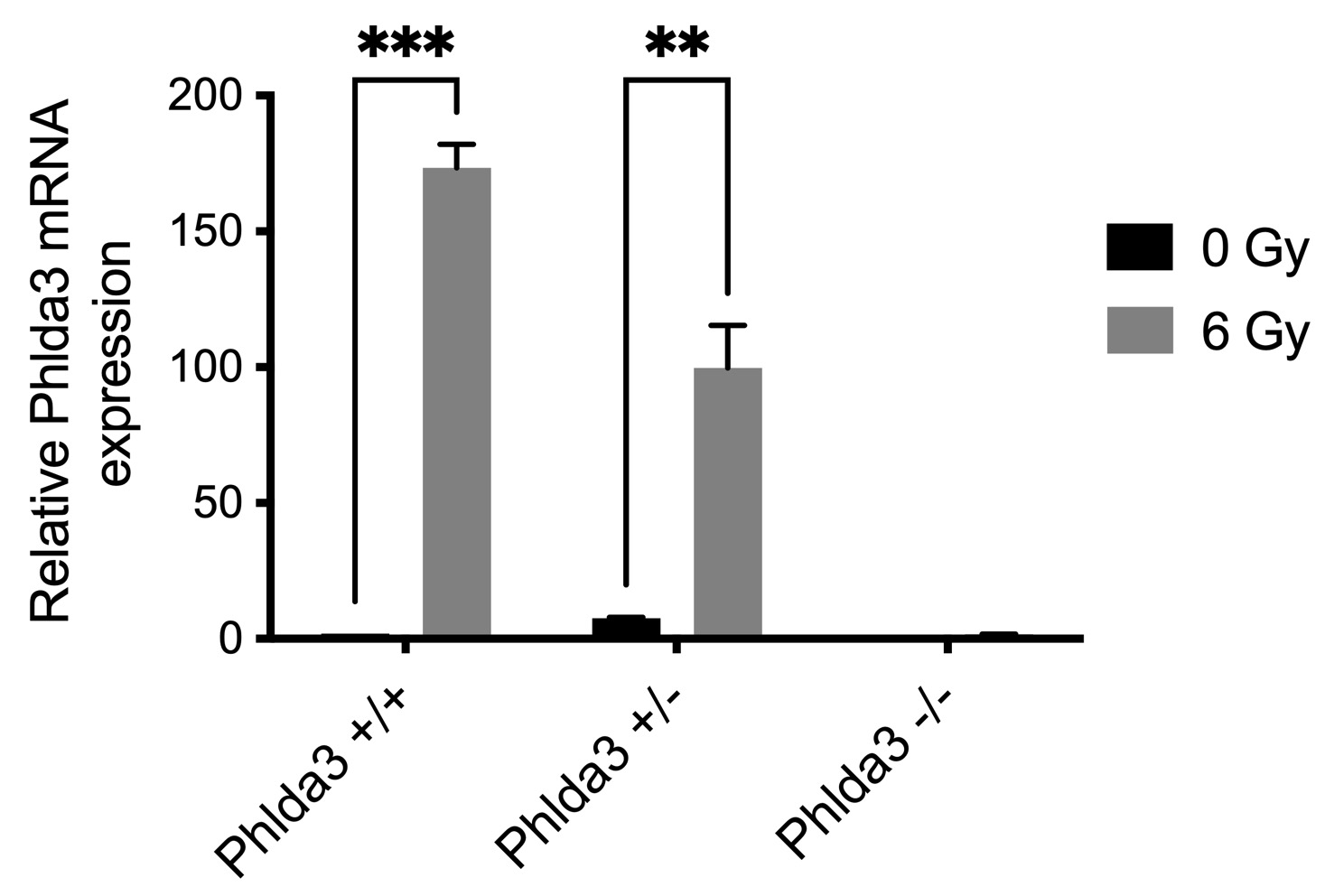


**Figure S5.** **Ionizing radiation induces *Phlda3* mRNA in mouse thymocytes.** Thymocytes harvested from *Phlda3^+/+^* , *Phlda3^+/-^* and *Phlda3^-/-^* mice were irradiated with 0 and 6 Gy X-rays *in vitro*. Cells were harvested 4 hours after irradiation to detect the expression of *Phlda3* mRNA. Data are presented as mean ± SEM. N=3 independent experiments. ***P<0.001 and **P<0.01 by Student’s t-test compared to 0 Gy.


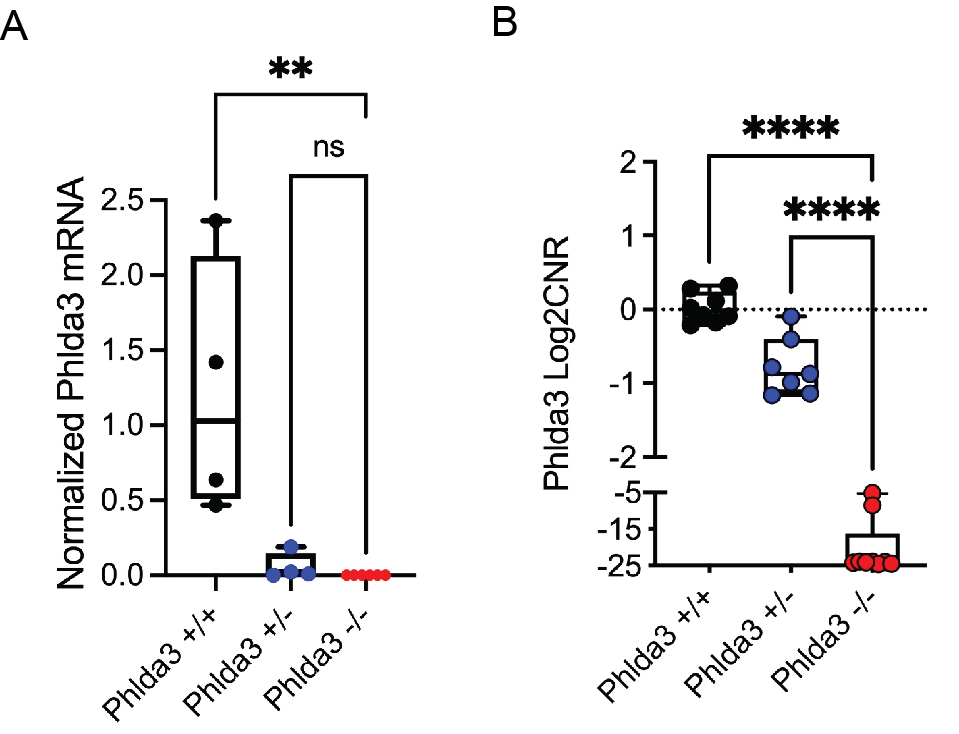


**Figure S6. Gene expression and copy number alterations of *Phlda3* in thymic lymphoma.** **A)** Expression of *Phlda3* mRNA in radiation-induced thymic lymphomas in *Phlda3^+/+^,* *Phlda3^+/-^* and *Phlda3^-/-^* mice. Each dot represents one tumor. Data are presented as mean ± SEM. **P<0.01 by one-way ANOVA with Bonferroni post-hoc test compared to *Phlda3^-/-^* tumors. **B)** The copy number (CNR) of *Phlda3* in radiation-induced thymic lymphomas in *Phlda3^+/+^,* *Phlda3^+/-^* and *Phlda3^-/-^* mice. Each dot represents one tumor. Data are presented as mean ± SEM. ****P<0.0001 by one-way ANOVA with Bonferroni post-hoc test compared to *Phlda3^-/-^* tumors.
